# Dual-modal radiomics ultrasound model to diagnose cervical lymph node metastases of differentiated thyroid carcinoma: a two-center study

**DOI:** 10.1186/s40644-025-00825-9

**Published:** 2025-01-20

**Authors:** Jiajia Tang, Yan Tian, Jiaojiao Ma, Xuehua Xi, Liangkai Wang, Zhe Sun, Xinyi Liu, Xuejiao Yu, Bo Zhang

**Affiliations:** 1 Department of Ultrasound, China-Japan Friendship Hospital, Beijing, China; 2 Department of Ultrasound, Peking Union Medical College Hospital, Beijing, China; 3 Chinese Academy of Medical Sciences and Peking Union Medical College, Beijing, China; 4 Capital Medical University, Beijing, China; 5 Department of Ultrasound, China-Japan Friendship Hospital, National Center for Respiratory Medicine, National Clinical Research Center for Respiratory Diseases, Institute of Respiratory Medicine of Chinese Academy of Medical Sciences, Beijing, China

**Keywords:** Differentiated thyroid carcinoma, Lymph node metastases, Ultrasound, Dual-modal radiomics, Machine learning, Feature extraction

## Abstract

**Objectives:**

To establish and validate a dual-modal radiomics nomogram from grayscale ultrasound and color doppler flow imaging (CDFI) of cervical lymph nodes (LNs), aiming to improve the diagnostic accuracy of metastatic LNs in differentiated thyroid carcinoma (DTC).

**Methods:**

DTC patients with suspected cervical LNs in two medical centers were retrospectively enrolled. Pathological results were set as gold standard. We extracted radiomic characteristics from grayscale ultrasound and CDFI images, then applied lasso (least absolute shrinkage and selection operator) regression analysis to analyze radiomics features and calculate the rad-score. A nomogram based on rad-score, clinical data, and ultrasound signs was developed. The performance of the model was evaluated using AUC and calibration curve. We also assessed the model’s diagnostic ability in European Thyroid Association (ETA) indeterminate LNs.

**Results:**

377 DTC patients and 726 LNs were enrolled. 37 radiomics features were determined and calculated as rad-score. The dual-modal radiomics model showed good calibration capabilities. The radiomics model displayed higher diagnostic ability than the traditional ultrasound model in the training set [0.871 (95% CI: 0.839–0.904) vs. 0.848 (95% CI: 0.812–0.884), *p*<0.01], internal test set [0.804 (95% CI: 0.741–0.867) vs. 0.803 (95% CI: 0.74–0.866), *p* = 0.696], and external validation cohort [0.939 (95% CI: 0.893–0.984) vs. 0.921 (95% CI: 0.857–0.985), *p* = 0.026]. The radiomics model could also significantly improve the detection rate of metastatic LNs in the ETA indeterminate LN category.

**Conclusions:**

The dual-modal radiomics nomogram can improve the diagnostic accuracy of metastatic LNs of DTC, especially for LNs in ETA indeterminate classification.

**Supplementary Information:**

The online version contains supplementary material available at 10.1186/s40644-025-00825-9.

## Introduction

Differentiated thyroid cancer (DTC) is the main type of increasing thyroid cancer. The incidence rate of metastatic lymph nodes (LNs) of DTC ranges from 20–50% [1]. Precise diagnosis of LNs preoperatively is of great importance in determining the scope of surgery and predicting the risk of DTC recurrence and metastasis. Ultrasound (US) is the first-line imaging technique in diagnosing thyroid and cervical LN disease, including grayscale ultrasound and color Doppler flow imaging (CDFI) [2–4]. However, preoperative ultrasound can only detect less than 30% of metastatic LNs, which may be related to the influence of trachea gas and the significant difference in experience of ultrasound physician [3]. Ultrasound-guided fine-needle aspiration (FNA) and thyroglobulin (Tg) tests are the main methods to confirm the diagnosis of metastatic LNs before surgery. However, FNA is invasive and requires operators to have high technical skills. Contrast-enhanced ultrasound is a complementary method, but the inconsistent signs of metastatic LNs reported in different studies make it difficult to be widely applied in clinical practice [4]. Lymphatic contrast-enhanced ultrasound is another emerging method. But it has not been widely used in routine practice, and its diagnostic accuracy needs to be further verified [5]. Due to the limited features captured by radiologist’s eyes, LNs diagnosis based on traditional signs (size, echogenecity, cystic change, calcification, et al. ) fails to make full use of the rich information in ultrasound images. It might result in low diagnostic accuracy and large differences among observers.

The intra- and interobserver variability remain the main problems faced in US examinations. LNs diagnosis based on US is subjective image interpretation, which is lack of effective quantification. Artificial intelligence (AI) technology represented by radiomics can mine high-throughput quantitative features from image data to reveal disease features [6]. We consider that radiomics based on machine learning is a potentially promising approach to extract quantitative features from US and CDFI images, enabling to add more measurable parameters and reduce demand for radiologist’s expertise in the diagnosis of DTC LNs. In predicting metastatic LNs of thyroid cancer, most of the studies extracted radiomics features from primary DTC lesions [7]. Those results cannot identify the specific location of the LNs, so they provide limited information for surgery. However, radiomics application in LN imaging is still rare. Only one study reported that the deep learning modal based on dual-modality US images showed systematically better accuracy, sensitivity, and specificity in the diagnosis of four common etiologies of unexplained cervical lymphadenopathy than skilled radiologists [3]. To the best of our knowledge, it has not been used in the characterization of metastatic LNs of DTC based on dual-modal ultrasound images yet.

Based on large-sample LN cases from two medical centers, this study aimed to extract and screen dual-modal radiomics information from grayscale ultrasound and CDFI images of DTC cervical LNs. A new model was established by combining radiomics scores, clinical information, and ultrasound signs. The diagnostic performance of the model was verified in two-center cases and compared with that of the traditional ultrasound model.

## Materials and methods

### Study cohorts

The ethics committee approved this retrospective, multicenter study, and written informed consent was exempted from the retrospective analysis. The checklist for evaluation of radiomics research was used as standards for reporting the process of study design, image acquisition, radiomics features extraction and model construction (Supplementary file 1). The sample size was calculated using PASS software using 5% margin of error and 90% confidence level. The sensitivity and specificity of diagnostic test one was 88.2% and 96.1%. The sensitivity and specificity of diagnostic test two was 75.5% and 66.4%. At least 216 LN cases were required for each diagnostic test, and a total of at least 432 LNs were included.

**Fig. 1:**
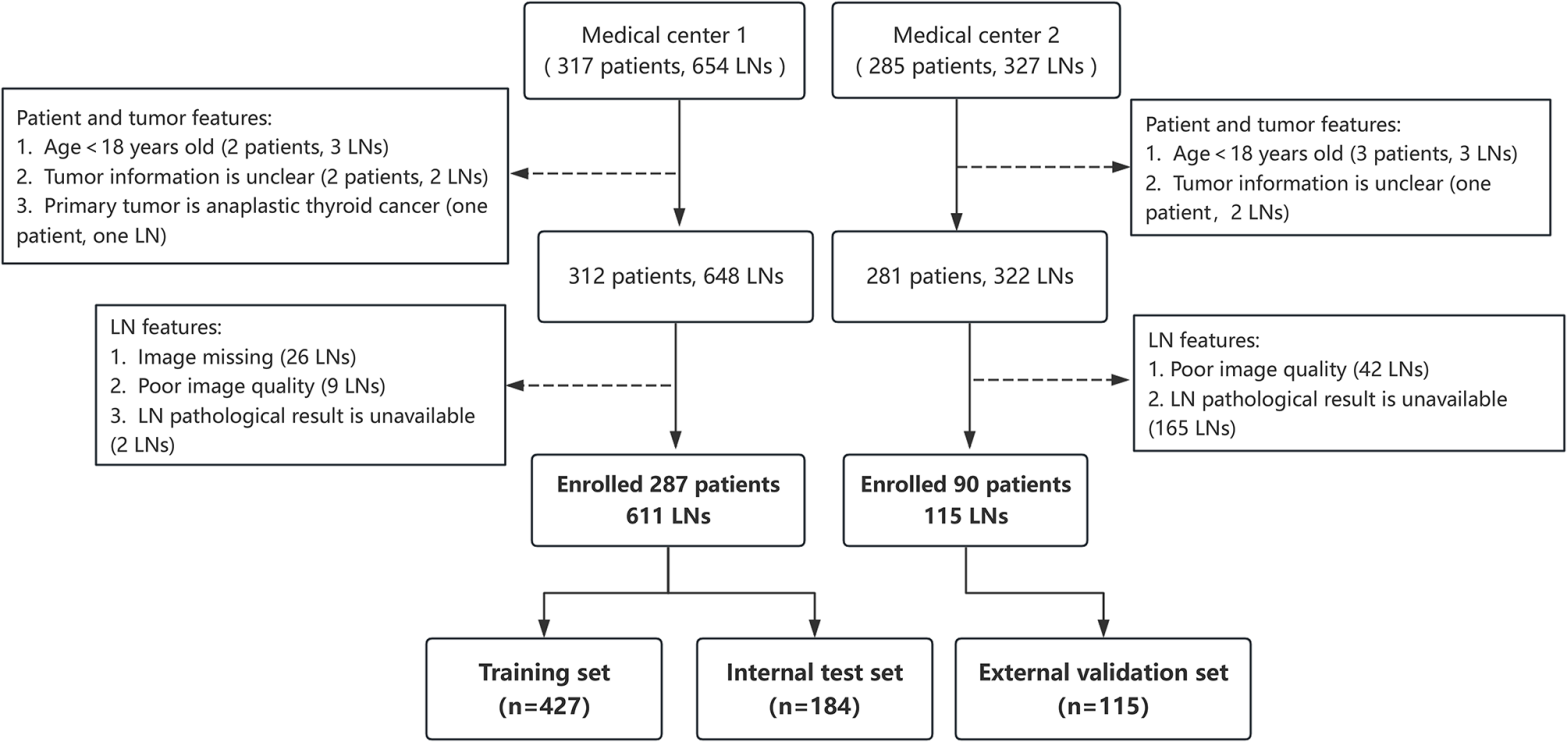
Schematic diagram of enrolling DTC LNs cases in two medical centers. DTC: differentiated thyroid cancer; LNs: lymph nodes

DTC patients with suspected LNs at the time of initial diagnosis or during cancer surveillance after surgery were collected from May 2022 to May 2023 at China-Japan Friendship Hospital (Medical Center 1). A similar enrollment process was performed from January 2017 to December 2018 at Peking Union Medical College Hospital (Medical Center 2). The inclusion criteria were as follows: (1) adult age; (2) planned for LNs fine-needle aspiration (FNA) or cervical LNs resection within one week after ultrasound examination; (3) complete ultrasound images were available, including grayscale ultrasound and CDFI images. The exclusion criteria were as follows: (1) having undergone previous radioactive iodine (^131^I) therapy; (2) the presence of other malignant tumors; (3) unclear pathological results and incomplete ultrasound images. The enrollment flowchart is shown in Fig. 1.

In this study, we used FNA cytological assessment and thyroglobulin in washout fluid or histological results from LN resection to confirm the diagnosis of LN. The criteria were as follows: (1) LNs were confirmed as benign if FNA cytological results showed no cancer cells and Tg was no more than 1 ng/mL in the washout fluid. If the postoperative pathology shows benign, it is confirmed to be benign. (2) LNs were confirmed to be metastatic if FNA cytological results indicated cancer cells, or Tg more than 10ng/ml. (3) LNs were confirmed as metastatic if the postoperative pathologic assessment showed metastasis of the resected LNs [4].

The participants in Medical Center 1 were randomly divided into the training and internal test cohorts at a ratio of 7:3 (R3.6.3: “createDataPartition”). The included cases in Medical Center 2 were set as the external validation cohort. Any part of the dataset in this study was not used in previous publication.

### Clinical information and ultrasound characteristics

**Fig. 2:**
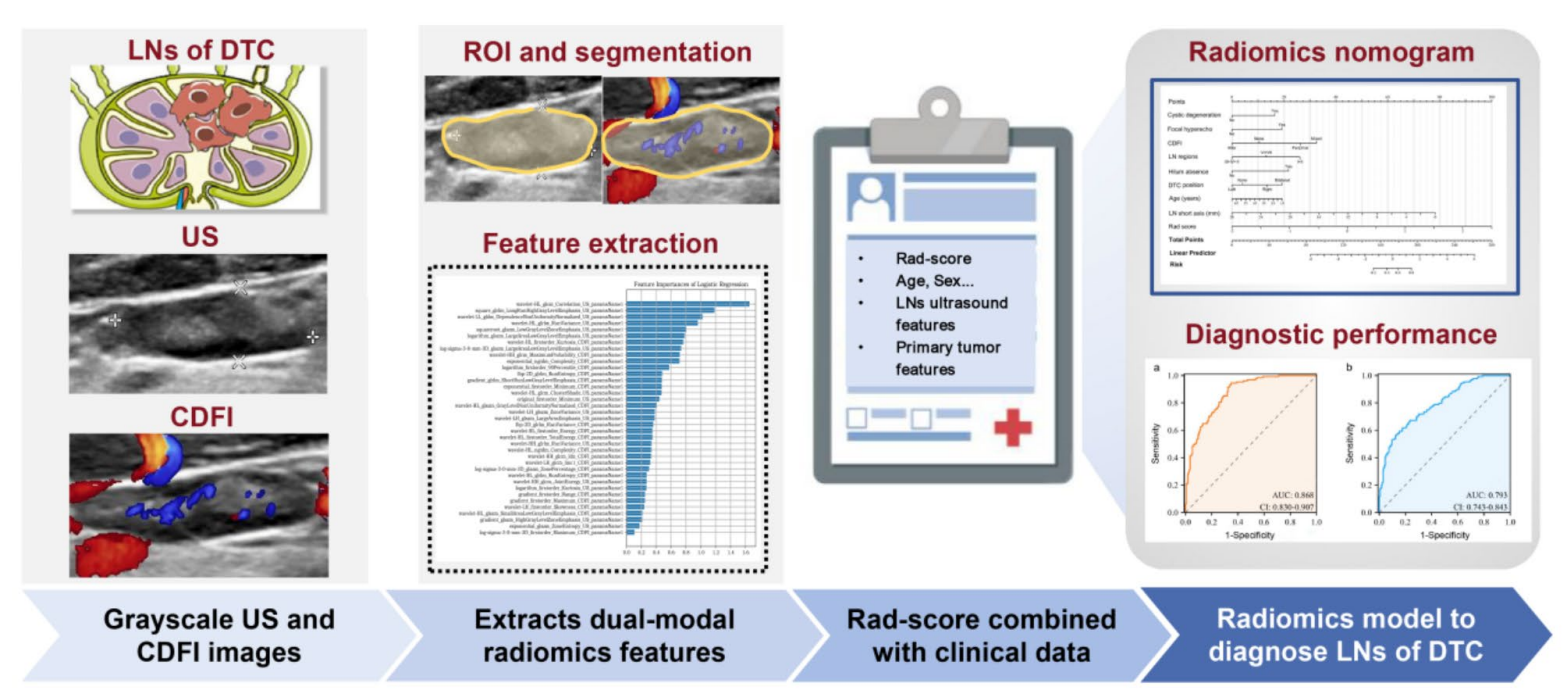
The workflow of the present study. LNs: lymph nodes; US: ultrasound; CDFI: color doppler flow imaging; Rad-score: radiomics score; ROI: region of interest; DTC: differentiated thyroid cancer

The key methological steps were summarized in Fig. 2. We evaluated and recorded patients’ basic information, LN information and primary tumor information according to published articles [5]. All included parameters were clinically important. We converted seven regions of LNs into three categories, including I + II, III + IV + V and VI + VII. None of the other indicators were converted. Patient age, gender, and other clinical information about DTC were respectively recorded. The data of the primary tumor included surgical treatment (none, partially or total thyroidectomy), the position of DTC (none, left lobe, right lobe, or bilateral lobes), foci of DTC (none, unifocal or multifocal), and the T status of the eighth edition American Joint Committee on Cancer (8th AJCC) [8].

In this study, an ultrasound examination was performed by a physician with 20 years of experience in thyroid ultrasound (linear array probe, 18-4 MHz or 12-5 MHz). Ultrasound machines included Philips EPIQ Elite, Philips EPIQ 7, and Philips iU22. Grayscale images were firstly acquired at the largest section of the LNs. More images were recorded for specific signs of LNs, such as focal hyperecho, cystic degeneration and microcalcifications. Then, the radiologists switched to the CDFI mode instantly and the scale of CDFI mode was 3 ~ 8 cm/s. The CDFI images were obtained at the section of LNs with most abundant blood flow signals. All the grayscale ultrasound and CDFI images of the same LNs were two-dimensional and stored in DICOM formats.

We assessed LN ultrasound images and collected the following information: LN location (median, left or right neck), LN level (I + II, III + IV + V, VI + VII), long/short axis (mm), round shape (long/short diameter < 2), hilum absence, focal hyperechogenicity, cystic degeneration, microcalcification, and CDFI features (no flow, hilar, peripheral or mixed flow). In addition, according to the LNs staging system published by the European Thyroid Association (ETA), all LNs can be classified into three categories: suspicious malignant, indeterminate, and normal LNs [9]. Diagnostic criteria for suspicious malignant LNs were the presence of any of the following: (1) focal hyperechogenicity, (2) cystic changes, (3) microcalcification, and (4) CDFI showed peripheral blood flow or increased diffuse blood flow. Normal LNs should meet the following four criteria: (1) remain normal hilum structure, (2) LNs were in oval shape and normal size (less than 8 mm in II level, less than 5 mm in III/IV levels), (3) no blood flow or hilar blood flow in CDFI (4) no signs of malignancy. The indeterminate LNs showed disappearance of hilum, and one of the following manifestations appears: (1) LNs are in round shape, (2) LNs in II level larger than 8 mm, and LNs in III/IV levels larger than 5 mm (3) CDFI detected increased blood flow.

The above ultrasound features and ETA staging of LNs were independently reviewed by two experienced radiologists who were blinded to the pathological results. In case of disagreement, a consensus was reached by the third radiologist.

### Dual-modal radiomics features from grayscale ultrasound and CDFI images

The Darwin research platform (https://arxiv.org/abs/2009.00908) was used to perform radiomic feature extraction and selection, which complies with the standardized processes and norms established by IBSI (Image Biomarker Standardization Initiative) for functions such as image data acquisition, preprocessing, feature extraction, as well as data processing methods [6]. We used manual methods to depict the region of interest (ROI) of grayscale ultrasound and CDFI images. As shown in Fig. 2, the boundaries of the ROI were determined by the visible contours of the LNs. The largest section of the grayscale ultrasound image was chosen for segmentation. One CDFI image with the most abundant blood flow signals was chosen for segmentation. Two radiologists (J.T.: 5 years of experience in ultrasound; Y.T.: 10 years of experience in ultrasound ) depicted the ROI of LNs on grayscale and CDFI images, respectively. For one image, each radiologist depicted the ROI three times. Then, the intraclass correlation coefficients (ICC) between and within two groups were calculated. Features with ICC ≥ 0.75 were retained. The final segmentation was based on the consensus of the two radiologists.

After segmentation, three groups of radiomic features were extracted: shape, first-order, and texture features. In short, shape features describe basic geometric characteristics, tumor size, shape, and surface roughness; first-order features describe the distribution of voxel intensities within the mask-defined image region using commonly used and basic metrics; texture features include gray-level co-occurrence matrices (GLCM), gray-level run-length matrices (GLRLM), gray-level size zone matrices (GLSZM), neighboring gray tone difference matrices (NGTDM), and gray-level dependence matrices (GLDM), which capture the spatial interdependence of voxels in images and show characteristics related to spatial heterogeneity such as gray change, granularity, and roughness of the image. As for image pre-processing, first-order and texture features were processed further using six filters, including exponential, square, square root, logarithm, log-sigma-3-0-mm-3D, and wavelet. Wavelet filters extracted features from the eight wavelet decomposition images. We performed resampling method (spline interpolation (order 3)). The physical parameters was 0.5 mm×0.5 mm. The image discretization method was binWidth = 25. After providing all modified parameters of pre-processing and radiomic feature extraction, all other parameters remained as a default configuration. We used the mean value-filled method to handle missing data. The min-max normalization technique was applied.

The Darwin research platform (https://arxiv.org/abs/2009.00908) with python backend was used for model development (Supplementary file 2). To reduce the complexity of the model, we first used ANOVA to screen the significance of the radiomics features. Those features with *p*-values less than 0.05 were included in the subsequent analysis. Then, we utilized the least absolute shrinkage and selection operator (lasso) method to screen out the radiomics features by optimizing the penalty parameter λ through the ten-fold cross-validation. Logistics regression analysis was used to calculate the rad-score.

### Construction and evaluation of dual-modal radiomics model

Rad-score, ultrasound features, and clinical information were included in the training set. We first used univariate logistics regression analysis to screen out the risk indicators related to DTC metastatic LNs (*p* < 0.05). Multivariate logistics regression analysis was subsequently used to determine the independent risk factors. Finally, the ultrasound radiomics model was established as a nomogram according to those independent risk variables. Meanwhile, ultrasound features and clinical information were integrated to establish a traditional ultrasound diagnostic model, which was compared with the new radiomics model.

The diagnostic performance of the two models was verified in the internal test set and external validation set. The diagnostic efficacy was assessed by calculating sensitivity, specificity, positive predictive value (PPV), negative predictive value (NPV), and accuracy. We depicted the receiver operating characteristic curve (ROC) and calculated the area under the curve (AUC). We also plotted the calibration curves, visually showing the comparison between the actual diagnostic performance and the model’s prediction performance. A decision curve analysis (DCA) was used to evaluate the clinical usefulness of the dual-modal radiomics nomogram by quantifying the net benefits.

### Statistical analysis

All statistical analyses were performed using R 3.6.3 software (http://www.r-project.org). Continuous variables were described using median and interquartile range (IQR). Categorical variables were described using frequency and percentage. We used the Chi-square test or Fisher’s exact test to compare differences in categorical variables. The independent-sample t-test was used for the normally distributed continuous variables. The Mann-Whitney U test was compared for the non-normally distributed continuous variables. Pairwise comparisons were performed using the significance test adjusted by Bonferroni. The inter-observer and intra-observer agreement was calculated with the kappa test. The maximum Youden index was used to define the optimal cut-off value of ROC.We used the DeLong test to compare the AUCs between the prediction models. A *p*-value less than 0.05 indicated a statistically significant difference.

## Results

### Basic information on patients and LNs

A total of 287 patients were included in medical center 1 (median age, 34 years [IQR, 30–42 years]; 224 women). All cases were pathologically diagnosed as papillary thyroid carcinoma. Of the 287 patients, 221 were untreated, and 66 had prior surgery with suspected LN recurrence. A total of 90 DTC patients were included in medical center 2 (median age, 37 years [IQR, 29–46 years], 63 women). Eighty-nine cases were confirmed as papillary thyroid cancer, and one case was follicular carcinoma. Of the 90 patients, 66 were untreated, and 24 had previously undergone surgical treatment. As shown in Table 1, there were no statistically significant differences in gender, age, characteristics of DTC, and history of surgical treatment between patients in two medical centers ( *p* > 0.05 ).

A total of 611 LNs were included in the medical center 1 (427 in the training set, 184 in the internal test set). There was no statistically significant difference in the ultrasound and pathological characteristics of LNs between the two groups ( *p*1 > 0.05 ) (Table 2). A total of 115 LNs were included in Medical Center 2 as an external validation set.

**Table 1:** The basic information of DTC patients

Variables	Medical center 1	Medical center 2	*p* value
**Patient number**	287	90	
**Gender**			0.118
Male	63 (22%)	27 (30%)	
Female	224 (78%)	63 (70%)	
**Age(years)**	34 (30, 42)	37 (29, 46)	0.120
**DTC position**			0.749
None	64 (22.3%)	24 (26.7%)	
Left lobe	91 (31.7%)	25 (27.8%)	
Right lobe	93 (32.4%)	27 (30%)	
Bilateral lobes	39 (13.6%)	14 (15.6%)	
**DTC foci**			0.270
None	64 (22.3%)	24 (26.7%)	
Single	174 (60.6%)	46 (51.1%)	
Multiple	49 (17.1%)	20 (22.2%)	
**AJCC T status**			0.673
T0	64 (22.3%)	24 (26.7%)	
T1	194 (67.6%)	60 (66.7%)	
T2	28 (9.8%)	6 (6.7%)	
T3	1 (0.3%)	0 (0%)	
**DTC surgical treatment**			0.543
None	221 (77%)	66 (73.3%)	
Partial thyroidectomy	24 (8.4%)	11 (12.2%)	
Total thyroidectomy	42 (14.6%)	13 (14.4%)	

DTC: differentiated thyroid carcinoma, AJCC: American Joint Committee on Cancer; T status: tumor status

**Table 2:** Ultrasound and pathological features of DTC suspicious LNs

Variables	Training set	Internal test set	p1	External validation	p2
**LN number**	427	184		115	
**LN location**			0.459		<0.001
Middle neck	9 (2.1%)	7 (3.8%)		52 (45.2%)	
Left neck	204 (47.8%)	89 (48.4%)		6 (5.2%)	
Right neck	214 (50.1%)	88 (47.8%)		57 (49.6%)	
**LN regions**			0.303		0.091
I + II	18 (4.2%)	5 (2.7%)		6 (5.2%)	
III + IV + V	260 (60.9%)	104 (56.5%)		57 (49.6%)	
VI + VII	149 (34.9%)	75 (40.8%)		52 (45.2%)	
**Size(mm)**					0.008
Long axis	10 (7, 13)	9 (7, 12)	0.093	12 (8, 15)	
Short axis	5 (4, 6)	4 (3.75, 6)	0.474	6 (5, 7.5)	
**Oval shape**			0.192		0.004
Yes	173 (40.5%)	85 (46.2%)		64 (55.7%)	
No	254 (59.5%)	99 (53.8%)		51 (44.3%)	
**Hilum absence**			0.971		<0.001
Yes	300 (70.3%)	129 (70.1%)		103 (89.6%)	
No	127 (29.7%)	55 (29.9%)		12 (10.4%)	
**Focal hyperecho**			0.235		0.001
Yes	122 (28.6%)	44 (23.9%)		51 (44.3%)	
No	305 (71.4%)	140 (76.1%)		64 (55.7%)	
**Cystic degeneration**			0.346		0.002
Yes	30 (7%)	17 (9.2%)		19 (16.5%)	
No	397 (93%)	167 (90.8%)		96(83.5%)	
**Microcalcification**			0.276		0.025
Yes	66 (15.5%)	35 (19%)		28 (24.3%)	
No	361 (84.5%)	149 (81%)		87 (75.7%)	
**CDFI**			0.574		0.002
None	148 (34.7%)	57 (31%)		23 (20%)	
Hilar	80 (18.7%)	43 (23.4%)		21 (18.3%)	
Peripheral	70 (16.4%)	31 (16.8%)		16 (13.9%)	
Mixed	129 (30.2%)	53 (28.8%)		55 (47.8%)	
**Pathological diagnosis**			0.967		<0.001
Malignant	205 (48%)	88 (47.8%)		76 (66.1%)	
Benign	222 (52%)	96 (52.2%)		39 (33.9%)	

DTC: differentiated thyroid carcinoma; CDFI: color doppler flow imaging;

*p*1: significance between training set and internal test set;*p*2 : significance between training set and external validation set

### Extraction and screening of radiomics features

For each LN, one grayscale ultrasound image and one CDFI image were selected to extract radiomics features. Both intra-observer ICC (range 0.802–0.977) and inter-observer ICC (range 0.781–0.982) performed well. Approximately 1125 features were extracted from grayscale ultrasound and CDFI images, respectively. In the training set, we utilized logistic regression analysis to assess the accuracy of grayscale ultrasound radiomics, CDFI radiomics, and the combined radiomics to diagnose metastatic LNs. The results showed that dual-modal radiomics features of grayscale ultrasound and CDFI had higher efficacy (Dual-modal US AUC: 0.707, CDFI AUC: 0.672, grayscale US AUC: 0.661). Therefore, radiomics information extracted from two ultrasound images was selected to establish a diagnostic model.

Firstly, 471 radiomics features with significant differences were screened from 2250 dual-modal ultrasound radiomics features (*p* < 0.05). Then, we used lasso regression analysis, and the penalty parameter λ was optimized by the ten-fold cross-validation method [λ: 0.708, log(λ): -0.155]. The optimal λ corresponded to 37 non-zero coefficients (radiomics features). Finally, we calculated the rad-score based on 37 radiomics features. The above radiomics characteristics and the calculated formula of rad-score were shown in Supplementary file 3.

### Development and validation of dual-modal radiomics model

**Fig. 3:**
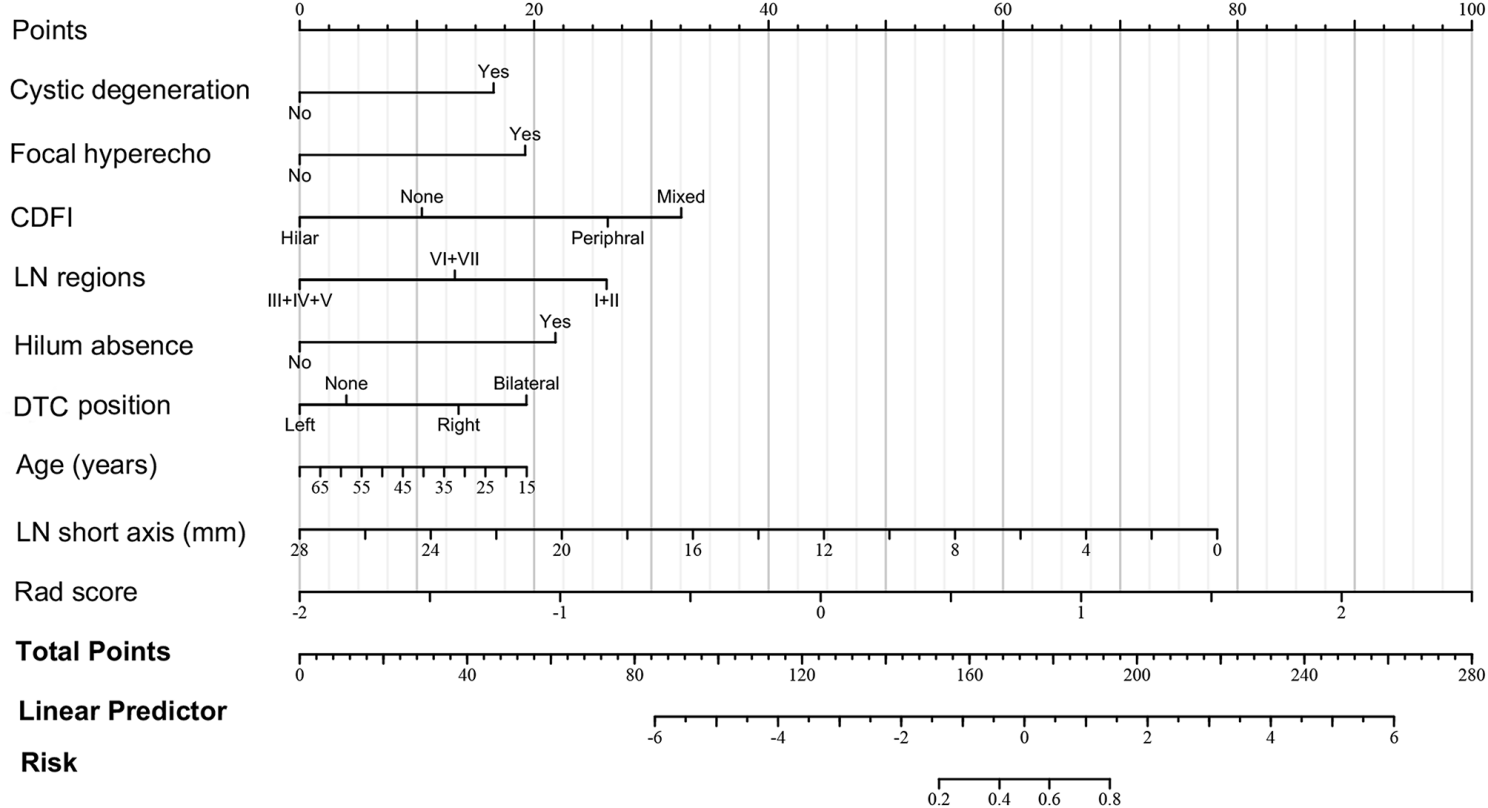
Radiomics nomogram of DTC metastatic LNs. For using the nomogram, values of each variable from a patient are located on each variable axis. A line is drawn upward to determine the points obtained for each variable on the point axis. The sum of these numbers is located on the total points axis. A line is drawn downward to the risk axis to determine the probability of metastatic LNs. DTC: differentiated thyroid cancer; LNs: lymph nodes; CDFI: color doppler flow imaging

We integrated rad-score, ultrasound signs, and LN clinical information to build the diagnostic model. Univariate logistics regression analysis was first performed, and stepwise regression was used to determine the factors significantly associated with metastatic LNs. Then, we performed a multivariate logistics regression analysis of the above factors. The final determined variables included patient age, DTC location, LN level, LN short axis, hilum absence, focal hyperechogenicity, cystic degeneration, CDFI signs, and rad-score (*p* < 0.001). Moreover, we established a radiomics nomogram for diagnosing DTC metastatic LNs (Table 3; Fig. 3). An example of nomogram application was shown in Supplementary file 4.

**Table 3:** Univariate and multivariate logistic regression analysis of the risk factors of metastatic LNs

Variables	Univariate logistics		β	Multivariate logistics	
	odds (95% CI)	*p* value		odds (95% CI)	*p* value
**Gender**					
Female	Reference			—	—
Male	1.169 (0.751–1.819)	0.489		—	—
**Age**	0.976 (0.958–0.994)	0.010*	-0.024	0.976 (0.952–1.002)	0.065*
**DTC position**					
Left lobe	Reference			Reference	
Right lobe	1.803 (1.091–2.981)	0.022*	0.921	2.512 (1.271–4.962)	0.008*
None	1.216 (0.734–2.014)	0.448	0.271	1.311 (0.672–2.558)	0.427
Bilateral lobes	2.741 (1.385–5.422)	0.004*	1.315	3.724 (1.504–9.220)	0.004*
**DTC foci**					
Single	Reference			—	—
Multiple	2.005 (1.131–3.555)	0.017*		—	—
None	0.877 (0.567–1.356)	0.555		—	—
**AJCC T status**					
T1	Reference				
T2	1.560 (0.935–2.601)	0.088		—	—
T3	3.360 (1.650–6.840)	<0.001		—	—
T0	1.055 (0.657–1.692)	0.825		—	—
**Thyroidectomy**					
Partial	Reference			—	—
Untreated	1.203 (0.629–2.298)	0.577		—	—
Total	0.895 (0.422–1.898)	0.772		—	—
**LN location**					
Right neck	Reference			—	—
Left neck	0.615 (0.418–0.905)	0.014*		—	—
Middle neck	1.076 (0.281–4.118)	0.915		—	—
**LN region**					
III + IV + V	Reference			Reference	
VI + VII	1.445 (0.965–2.166)	0.074	0.899	2.458 (1.393–4.337)	0.002*
I + II	2.561 (0.933–7.034)	0.068	1.779	5.925 (1.347–26.076)	0.019*
**Oval shape**					
No	Reference			—	—
Yes	1.037 (0.705–1.527)	0.852		—	—
**Hilum absence**					
No	Reference			Reference	
Yes	0.247 (0.156–0.392)	< 0.001*	-1.483	0.227 (0.121–0.427)	< 0.001*
**Cystic degeneration**					
No	Reference			Reference	
Yes	3.881 (1.628–9.253)	0.002*	1.124	3.078 (0.964–9.826)	0.058*
**Focal hyperechogenicity**					
No	Reference			Reference	
Yes	2.884 (1.858–4.476)	<0.001*	1.307	3.696 (2.021–6.760)	< 0.001*
**CDFI**					
Hilar	Reference			Reference	
None	1.518 (0.798–2.886)	0.203	0.709	2.031 (0.870–4.741)	0.101
Mixed	11.715 (5.990–22.914)	<0.001*	2.212	9.138 (4.013–20.808)	< 0.001*
Peripheral	7.573 (3.642–15.744)	<0.001*	1.786	5.968 (2.388–14.915)	< 0.001*
**Microcalcification**					
No	Reference				
Yes	1.828 (1.070–3.122)	0.027*		—	—
**LN long axis**	1.014 (0.981–1.047)	0.418		—	—
**LN short axis**	1.059 (0.975–1.150)	0.173	-0.190	0.827 (0.732–0.934)	0.002*
**Radiomics score**	4.759 (3.186–7.108)	< 0.001*	1.510	4.528 (2.564–7.997)	< 0.001*

LN: lymph node; CDFI: color doppler flow imaging; DTC: differentiated thyroid carcinoma; CI: confidential interval; AJCC: American Joint Committee on Cancer; T status: tumor status

**Fig. 4:**
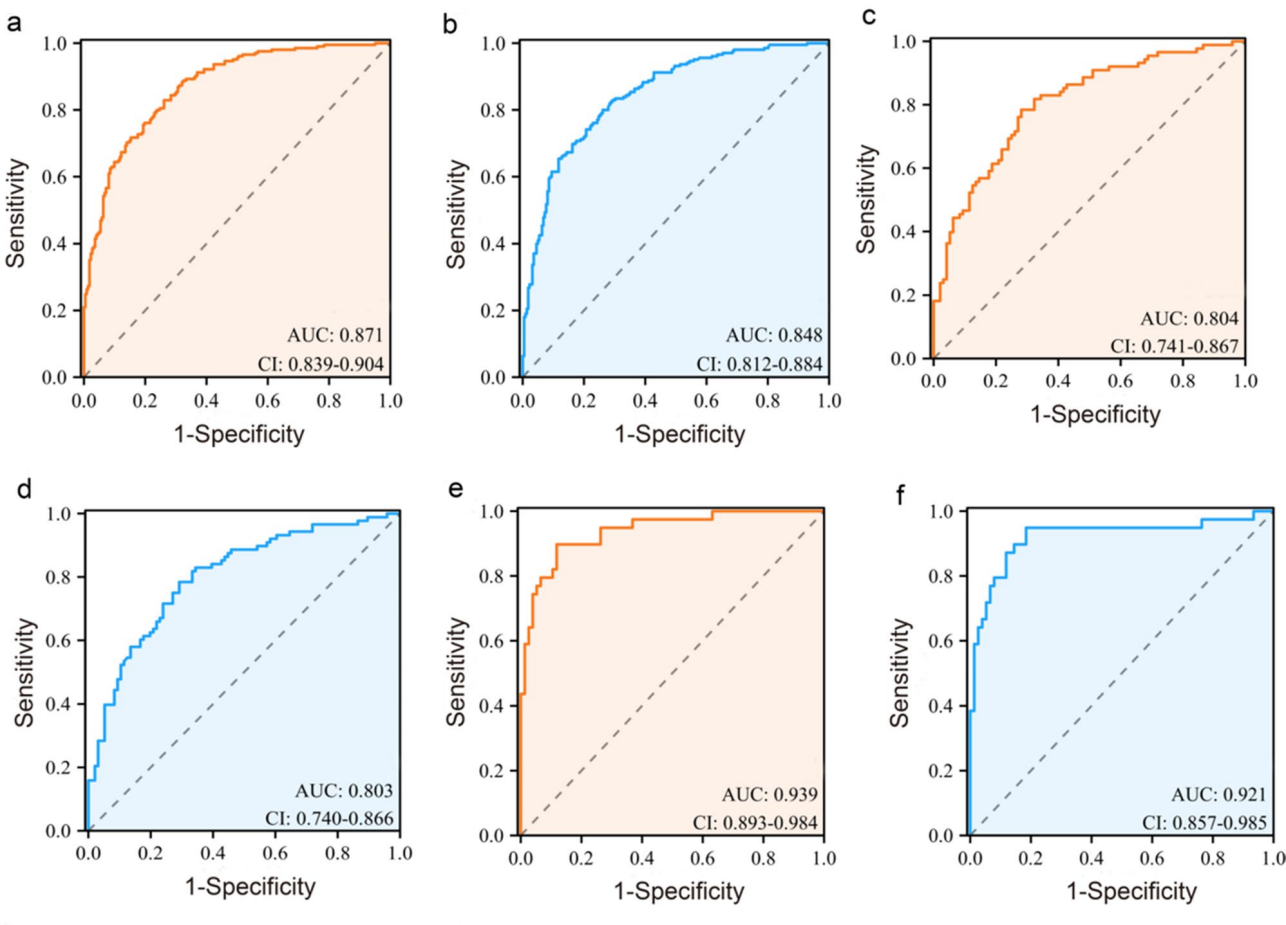
Diagnostic efficacy of radiomics and traditional US models in the (**a**, **b**) training set, (**c**, **d**) internal test set and (**e**, **f**) external validation set. AUC: area under the receiver operating characteristic curve, CI: confidence interval; US: ultrasound

**Fig. 5:**
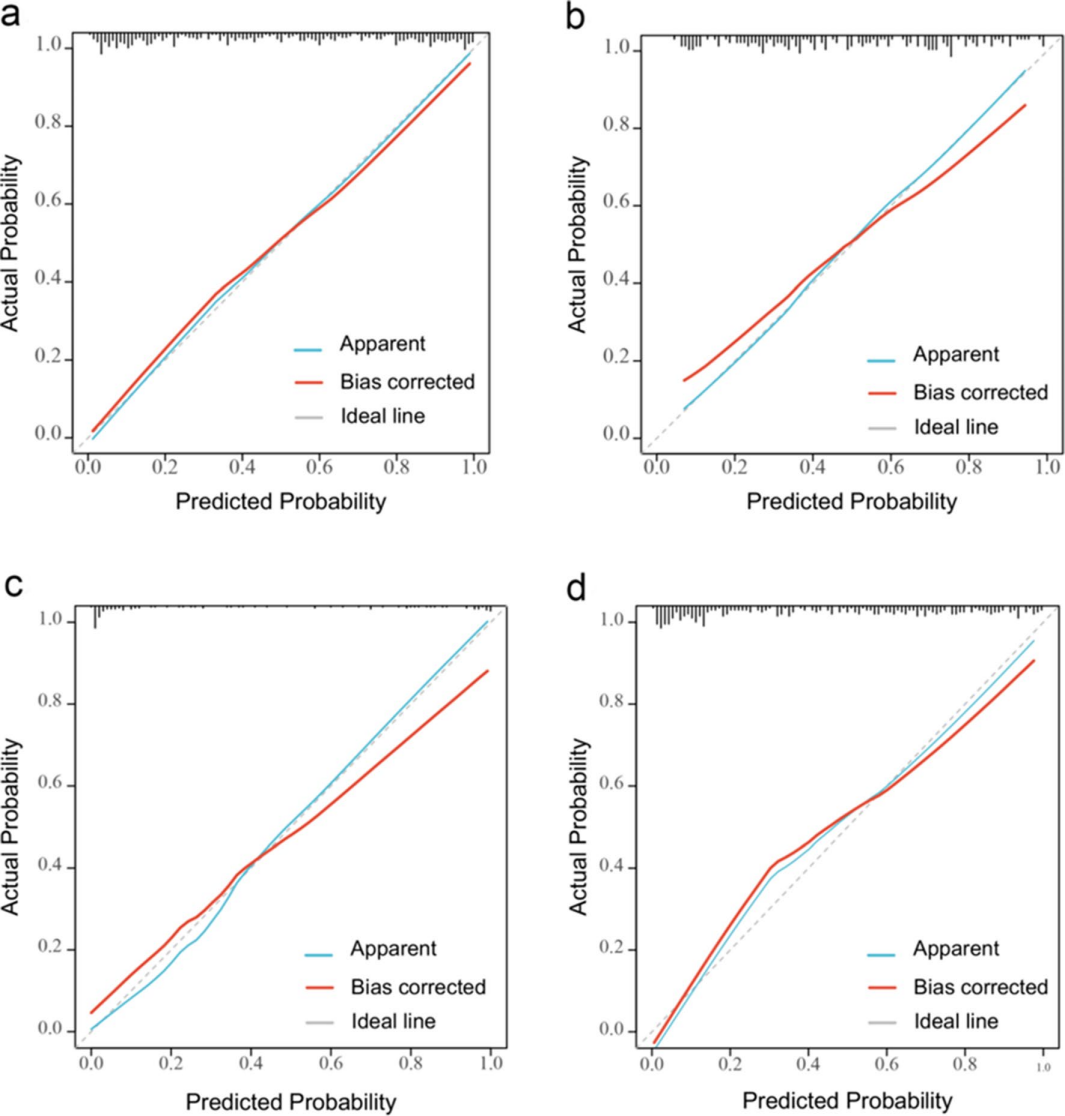
Calibration curves of the radiomics model in (**a**) training set, (**b**) internal test set and (**c**) external validation set. (**d**) Calibration curve of radiomics model for diagnosing ETA indeterminate LNs. ETA: European Thyroid Association; LNs: lymph nodes

**Fig. 6:**
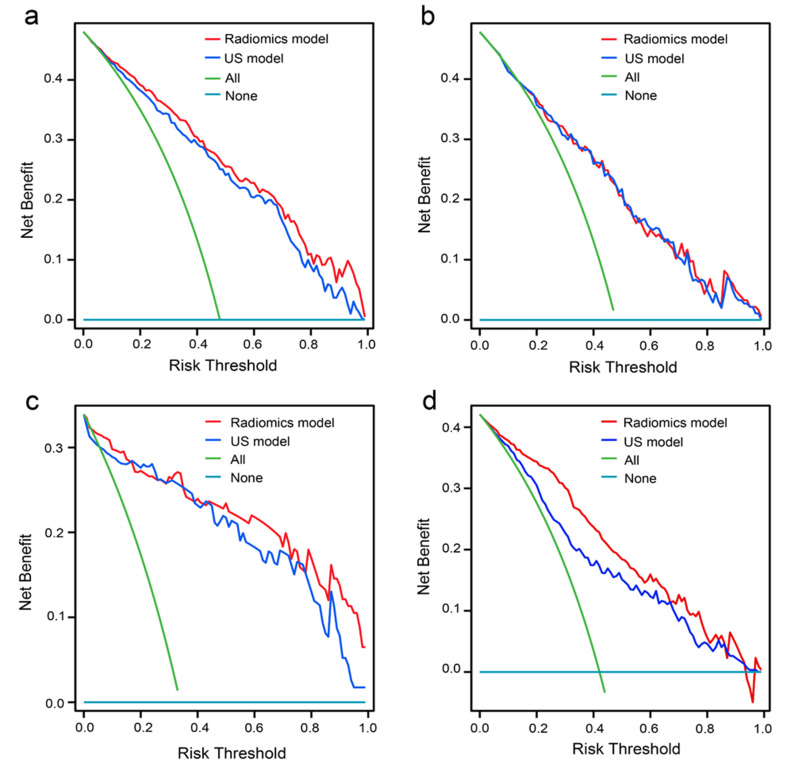
Decision curve analysis (DCA) of two models in predicting metastatic LNs derived from (**a**) training set, (**b**) internal test set and (**c**) external validation set. (**d**) DCA of two models for diagnosing metastatic LNs in ETA indeterminate LNs. ETA: European Thyroid Association; LNs: lymph nodes; US: ultrasound

The radiomics model displayed higher diagnostic accuracy than the traditional ultrasound model in the training set, internal test set, and external validation set (Table 4). The AUC of the radiomics and traditional ultrasound models in the training set was 0.871 (95%CI: 0.839–0.904) and 0.848 (95%CI: 0.812–0.884), respectively. The *p*-value of the DeLong test was less than 0.001 (Fig. 4a, b). The AUC of the two models in the internal test set was 0.804 (95% CI: 0.741–0.867) and 0.803 (95% CI: 0.74–0.866) with a *p*-value of 0.696 (Fig. 4c, d). In the external validation set, the radiomics model showed significantly higher diagnostic efficacy than the traditional ultrasound model [(AUC 0.939 (95%CI: 0.893–0.984) vs. 0.921 (95%CI: 0.857–0.985)] and the *p*-value between two models was 0.026 (Fig. 4e, f). In addition, the calibration curves showed that the radiomics model had a good degree of calibration (Fig. 5a-c).The DCA showed that the radiomics nomogram was more beneficial than the US model in the training and external validation sets (Fig. 6a, c). In the internal test set, the clinical net benefit was similar for two models (Fig. 6b).

**Table 4:** The diagnostic performance of radiomics and traditional US models for DTC metastatic lymph nodes

	Sensitivity (%)	Specificity (%)	PPV(%)	NPV (%)	Accuracy (%)
**Training set**					
Radiomics model	82.9 (79.4–85.9)	73.9 (69.9–77.6)	74.6 (70.6–78.2)	82.4 (78.8–85.5)	78.2 (74.4–81.6)
US model	80 (76.3–83.3)	73.9 (69.9–77.6)	73.9 (69.9–77.6)	80 (76.3–83.3)	76.8 (72.9–80.3)
**Internal test set**					
Radiomics model	78.4 (73.4–82.7)	71.9 (66.5–76.7)	71.9 (66.6–76.7)	78.4 (73.4–82.7)	75 (69.8–79.6)
US model	78.4 (73.4–82.7)	70.8 (65.4–75.6)	71.1 (65.7–75.9)	78.2 (73.2–82.5)	74.5 (69.3–79.2)
**External validation set**					
Radiomics model	89.7 (84.7–93.2)	88.2 (83.0–92.0)	79.5 (73.4–84.7)	94.4 (90.2–96.9)	88.7 (83.6–92.4)
US model	94.9 (90.5–97.4)	86.1 (80.5–90.4)	72.5 (65.9–78.3)	96.9 (93.4–98.6)	86.1 (80.5–90.4)

The data in brackets represent the 95% confidence intervals. US: Ultrasound; DTC: differentiated thyroid carcinoma; PPV: positive predictive value; NPV: negative predictive value

### Diagnostic performance of radiomics model for ETA indeterminate LNs

**Fig. 7:**
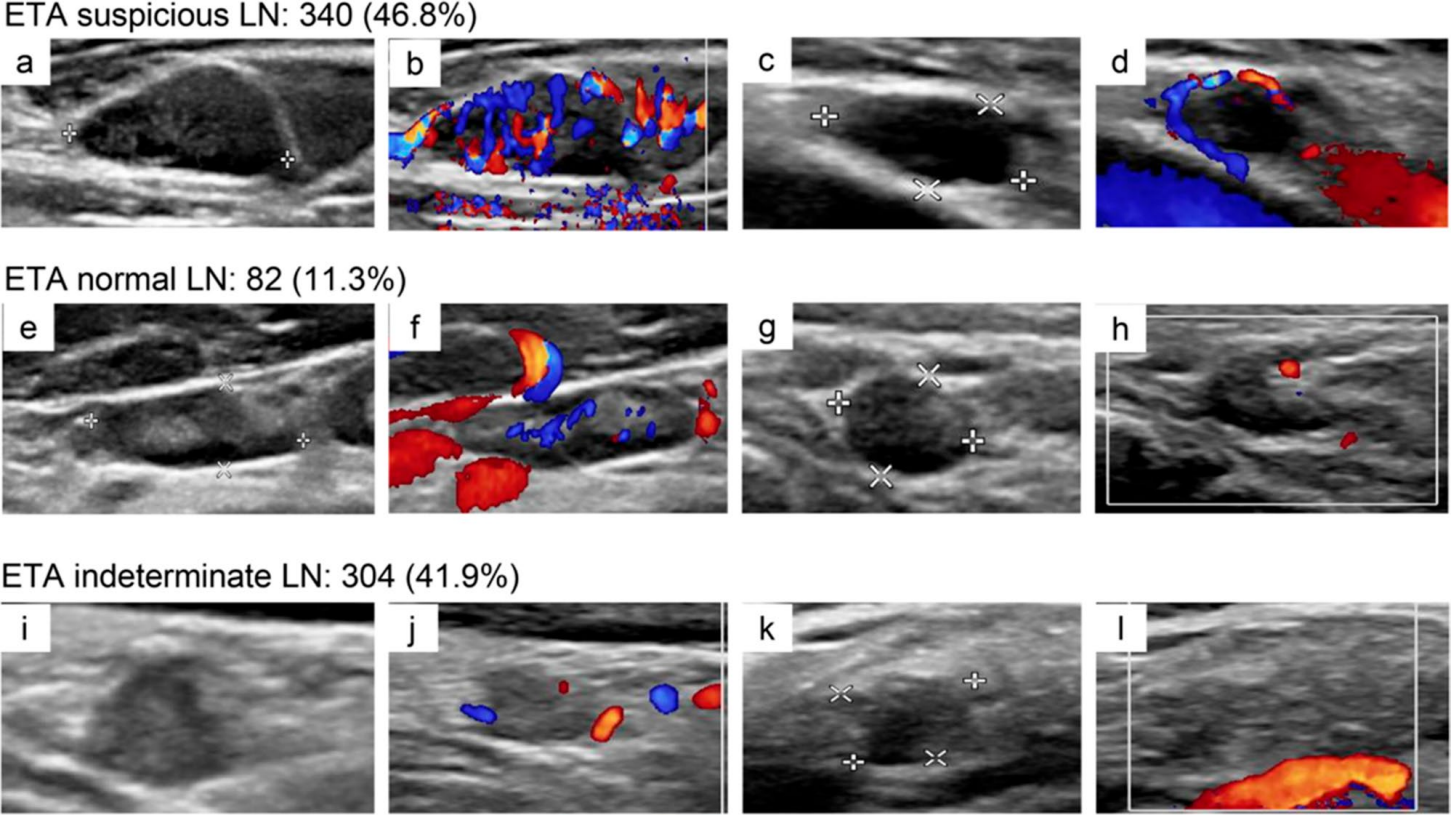
Examples of LNs in different ETA classifications. (**a**-**d**) ETA suspicious malignant LNs: (**a**, **b**) Hilum disappears with focal hyperechoic, and CDFI shows mixed and rich blood flow; (**c**, **d**) Hilum disappears and CDFI shows peripheral blood flow; (**e**-**h**) ETA normal LNs: (**e**, **f**) Hilum is clearly detected and CDFI shows hilar blood flow; (**g**, **h**) LN cortical is thickening with normal hilum and CDFI shows punctate blood flow. (**i**-**l**) ETA indeterminate LNs: (**i**, **j**) Hilum is unclear and CDFI shows punctate blood flow; (**k**, **l**) Hilum is unclear and no blood flow signal in CDFI. LNs: lymph nodes; CDFI: color doppler flow imaging; ETA: European Thyroid Association

According to ETA criteria, 340 cases (46.8%) were classified as ETA suspicious malignant LNs in this study (Fig. 7a-d), of which 104 cases were pathologically benign LNs and 236 were metastatic LNs. The malignancy rate was 69%. A total of 82 cases (11.3%) were classified as “normal LNs” (Fig. 7e-h), of which 77 cases were truly benign LNs and 5 cases were metastatic LNs. The malignancy rate of the normal LN category was 5.1%. A total of 304 cases (41.9%) were classified as “indeterminate LNs” in the ETA classification (Fig. 7i-l). Among indeterminate LNs, 176 were truly benign, and 128 cases were pathological LN metastases, with a malignancy rate of 42.1%. The results indicated that, according to our data, the proportion of metastatic LNs in “ETA indeterminate LN” was still high.

**Fig. 8:**
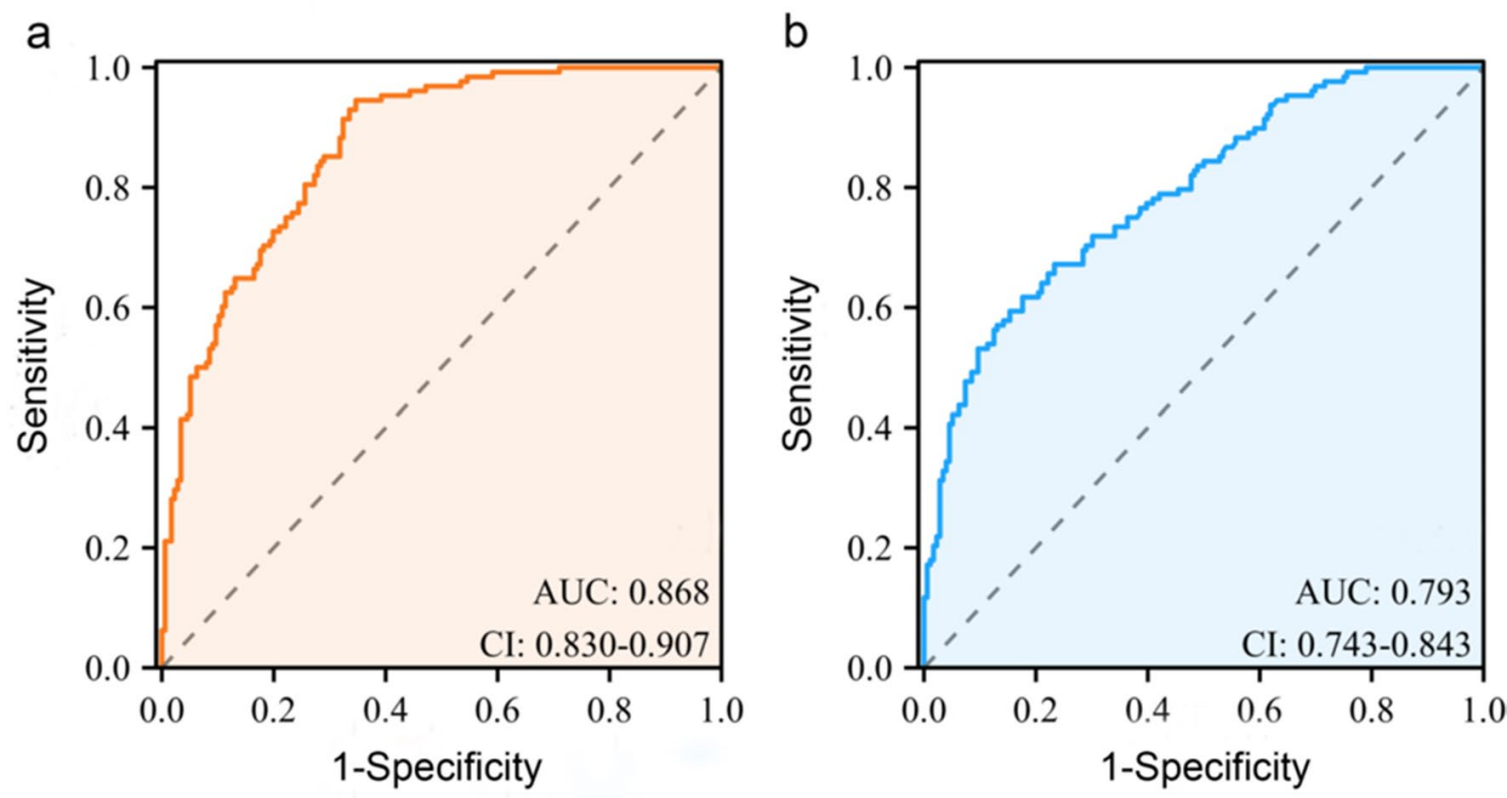
The diagnostic performance of (**a**) radiomics model and (**b**) traditional ultrasound model for ETA indeterminate LNs. ETA: European Thyroid Association; AUC: area under the receiver operating characteristic curve; CI: confidence interval

In this study, 304 indeterminate LNs were brought into the radiomics model and traditional ultrasound model for subgroup analysis. The results showed that the radiomics model could significantly improve the sensitivity and NPV of metastatic LNs in the ETA indeterminate LN category (Table 5). The AUCs of the two models were 0.868 (95%CI: 0.830–0.907) and 0.793 (95%CI: 0.743–0.843), respectively. The *p*-value of the DeLong test between the two models was less than 0.001 (Fig. 8). In addition, calibration plots demonstrated the optimal consistency between the bootstrap-predicted values and the actual observed values, indicating the appreciable reliability of the radiomics model (Fig. 4d). The DCA showed that the radiomics nomogram was more beneficial than the US model for identifying metastasis in ETA indeterminate LNs (Fig. 6d).

**Table 5:** The diagnostic performance of radiomics and traditional US models for ETA indeterminate lymph nodes

	Sensitivity (%)	Specificity (%)	PPV (%)	NPV (%)	Accuracy (%)
Radiomics model	94.5 (90.2–96.9)	65.3 (59.6–70.6)	66.5 (60.8–71.8)	94.3 (89.8–96.8)	77.6 (72.1–82.3)
US model	61.7 (55.6–67.5)	82.4 (77.0-86.7)	71.8 (65.4–77.4)	74.7 (68.7–79.9)	73.7 (67.8–78.9)

The data in brackets represent the 95% confidence intervals. US: Ultrasound; ETA: European Thyroid Association; PPV: positive predictive value; NPV: negative predictive value

## Discussion

DTC accounts for more than 90% of all thyroid cancers [10]. LNs metastasis is one of the most significant variables to determine the prognosis of DTC. However, ultrasound examinations, consisting of conventional ultrasound and CEUS, still plays a limited role in detecting metastatic DTC [11–14]. In our study, we extracted multiple radiomics features from grayscale ultrasound and CDFI images of LNs. Moreover, we constructed a dual-modal radiomics nomogram, which showed better performance in identifying metastatic LNs than traditional ultrasound model.

In recent years, application of AI in diagnostic studies has been a frontier direction [15]. Zhu et al. enrolled a large sample of patients from three medical centers and built a deep-learning model based on grayscale ultrasound and CDFI images to distinguish unexplained enlarged LNs [3]. Their results showed that malignant LNs were often associated with unique features, including extensive structural variation (intranodal necrosis) and abundant peripheral blood vessels [16]. These findings were similar with the results of our study. In contrast, reactive proliferative LNs often had no vascular or hilar blood flow [17]. Unlike Zhu et al.‘s research, our study included multiple factors such as clinical information, primary tumor features, and LN ultrasound signs. Our results showed that age, DTC location, LN levels, and various LN ultrasound signs were related to metastatic LNs. The new model, which combined Rad-score with the above factors, demonstrated higher diagnostic performance and was easier to apply to clinical practice. In addition, we applied the radiomics model to LNs which were difficult to diagnose as benign or metastatic by ultrasound. The sensitivity and NPV of the new model were obviously improved, showing higher application value in clinical practice.

ETA has developed a classification system based on the ultrasound characteristics of cervical LNs [9]. Yoo et al. found that among 384 LNs, 194 (55.7%), 72 (20.7%), and 82 (23.6%) LNs were categorized as ETA suspicious malignant LNs, normal LNs, and indeterminate LNs, respectively [18]. In our study, 340 (46.8%) cases were ETA suspicious LNs, 82 (11.2%) were normal LNs, and 304 (41.9%) were indeterminate LNs. Chung et al. showed that among the 236 cases of ETA indeterminate LNs, 67 cases (28.3%) were pathologically metastatic LNs [19]. Our study showed a higher proportion of metastatic LNs in ETA indeterminate LNs [42.1% (128/304)], which indicated difficulty in identifying metastatic LNs according to traditional ultrasound characteristics. And there is no better way to improve the preoperative detection rate of these metastatic LNs. In response to this problem, researchers have carried out various explorations. For instance, Lee et al. combined super-microvascular imaging (SMI) with conventional ultrasound to establish an SMI-based LN grading system [20]. The system’s sensitivity, specificity, and accuracy were 78.1%, 94.9%, and 86.3%. However, there were still significant observer differences in the characteristics of SMI, and SMI had yet to be widely used in clinical practice. Also, the sample size of this study was small, so the diagnostic ability of SMI for metastatic LNs needs to be further verified.

In this study, we established a new model to diagnose metastatic LNs by combining clinical information, ultrasound signs, and rad-score. For ETA indeterminate LNs, this radiomics nomogram could significantly improve the sensitivity and NPV of metastatic LNs. The reason is that machine learning techniques can achieve automatic extraction and quantification of image features imperceptible by naked eyes. Therefore, we believe that the high throughput of radiomics information can compensate for the shortcomings of traditional ultrasound features. Moreover, the radiomics nomogram undoubtedly play a potential role in guiding radiologists, especially when they are facing challenging in diagnosing some indeterminate LNs. In clinical practice, the application of this radiomics model may reduce the rate of missed diagnosis of metastatic LNs and minimize inappropriate treatment. Nevertheless, it also needs to be admitted that the CIs of AUC for the radiomics model and the traditional ultrasound model overlap significantly in both the training and validation cohorts. This may be due to the fact that the radiomics features are derived from grayscale ultrasound and CDFI images of LNs, so there is a feature overlap between radiomics features and the original images. In addition, the radiomics nomogram also contains traditional ultrasound parameters of LN and DTC, which resulting in overlapping between two models. As for the diagnostic efficacy of the radiomics model, we found the external validation cohort outperformed the internal test set (Table 4**)**. The possible reason might be that all the LN cases in external validation cohorts were undergone cervical LN resection. For those LNs which were decided to have surgical dissection, their malignant ultrasound features were more pronounced, so the diagnostic accuracy of metastatic LNs was relatively higher when the radiomics model was applied.

In summary, the radiomics model has significantly improved sensitivity and NPV for identifying metastatic LNs in ETA indeterminate LNs, which is essential to minimize missed metastatic LNs. It is of note that radiomics features and the calculated Rad-score have a critical contribution to the new model. It is undeniable that the radiomics model shows relatively low specificity and PPV, which may lead to an increase in false positive LNs and overtreatment. This study still had some limitations. Firstly, the retrospective nature of this study caused inevitable deviations. The generalizability of the model should be further verified in a prospective cohort. Secondly, the selected grayscale ultrasound and CDFI were static images, and the extracted information was inevitably limited. Moreover, the established model was comprehensive, combining radiomics with clinical and ultrasound features. The efficacy of radiomics information alone in diagnosing metastatic LNs was low. We need to conduct subsequent studies to explore more suitable algorithms for imaging feature extraction.

## Conclusions

This study determined specific radiomics features for diagnosing DTC metastatic LNs based on grayscale ultrasound and CDFI images. The dual-modal radiomics model showed higher diagnostic accuracy than the traditional ultrasound model. For indeterminate LNs in the ETA system, the new model could improve the diagnostic sensitivity of metastatic LNs, and minimize the missed metastatic LNs.

## Electronic supplementary material

Below is the link to the electronic supplementary material.

Supplementary Material 1

Supplementary Material 2

Supplementary Material 3

Supplementary Material 4

## Data Availability

No datasets were generated or analysed during the current study.

## Abbreviations

AJCC: American Joint Committee on Cancer
AI: Artificial intelligence
AUC: Area under the receiver operating curve
CDFI: Color Doppler flow imaging
CEUS: Contrast-enhanced ultrasound
CI: Confidence interval
DTC: Differentiated thyroid cancer
ETA: European Thyroid Association
FNA: Fine-needle aspiration
ICC: Intraclass correlation coefficient
IQR: Interquartile range
lasso: Least absolute shrinkage and selection operator
LN: Lymph node
NPV: Negative predictive value
PPV: Positive predictive value
ROI: Region of interest
Tg: Thyroglobulin
US: Ultrasound
