# Supplementary material for: Dual-modal radiomics ultrasound model to diagnose cervical lymph node metastases of differentiated thyroid carcinoma: a two-center study
Source: Cancer Imaging. 2025 Jan 20;25:4. doi: 10.1186/s40644-025-00825-9 (PMC11749166; doi:10.1186/s40644-025-00825-9)
Supplement: Supplementary file 2 — Supplementary Material 2 [file 40644_2025_825_MOESM2_ESM.docx]

**Algorithm details:**

The Darwin research platform (<https://arxiv.org/abs/2009.00908>) with python backend.

sklearn.feature_selection.SelectKBest(f_classif,k)

sklearn.feature_selection.SelectFromModel(LogisticRegressionCV(penalty='l1'), threshold=None, prefit=False, norm_order=1, max_features=None)

sklearn.linear_model.LogisticRegression()

**Training and tuning details:**

1. Tolerance for stopping criteria: 0.0001.(i,e., if the updates are smaller than this value, we assume the model has converged and stop training)
2. optimization techniques：Stochastic Average Gradient Accelerated Method

regularization parameters：elasticnet; l1_ratio: 0.5

1. initialization of model parameters：No
2. hyperparameter tuning strategy：No
3. range of hyperparameter：No

**Handling of confounders:**

Combat normalization was used to address confounding
