# Supplementary material for: Dual-modal radiomics ultrasound model to diagnose cervical lymph node metastases of differentiated thyroid carcinoma: a two-center study
Source: Cancer Imaging. 2025 Jan 20;25:4. doi: 10.1186/s40644-025-00825-9 (PMC11749166; doi:10.1186/s40644-025-00825-9)
Supplement: Supplementary file 3 — Supplementary Material 3 [file 40644_2025_825_MOESM3_ESM.docx]

**Supplementary file 3**


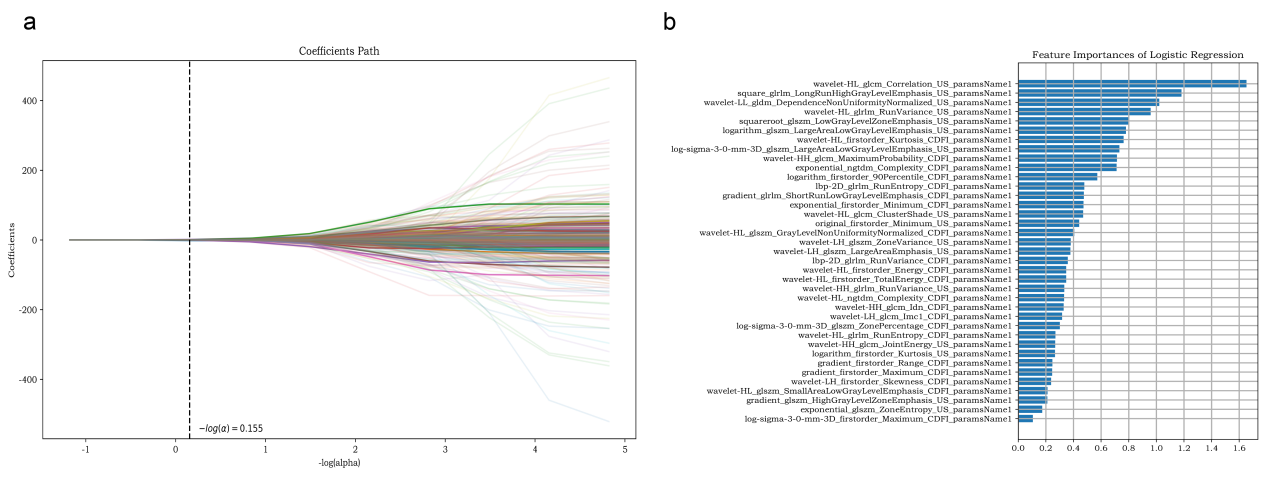


**Supplementary Figure 3. Radiomics feature selection using lasso regression model in the training cohort.** (**a**) The vertical line was drawn at the value selected using 10-fold cross-validation, where optimalλresulted in 37 non-zero coefficients. (**b**) 37 radiomics features and weight coefficients. The ordinate shows the name of the ultrasound radiomics features and the abscissa is the weight coefficient. lasso, least absolute shrinkage and selection operator.

The Rad-score was calculated as follows:

Rad-score =

-1.653*wavelet-HL_glcm_Correlation_US_paramsName1

-1.182*square_glrlm_LongRunHighGrayLevelEmphasis_US_paramsName1

+1.021*wavelet-LL_gldm_DependenceNonUniformityNormalized_US_paramsName1

+0.959*wavelet-HL_glrlm_RunVariance_US_paramsName1

+0.802*squareroot_glszm_LowGrayLevelZoneEmphasis_US_paramsName1

+0.780*logarithm_glszm_LargeAreaLowGrayLevelEmphasis_US_paramsName1

-0.763*wavelet-HL_firstorder_Kurtosis_CDFI_paramsName1

+0.733*log-sigma-3-0-mm-3D_glszm_LargeAreaLowGrayLevelEmphasis_US_paramsName1

-0.715*wavelet-HH_glcm_MaximumProbability_CDFI_paramsName1

+0.712*exponential_ngtdm_Complexity_CDFI_paramsName1

+0.573*logarithm_firstorder_90Percentile_CDFI_paramsName1

+0.479*lbp-2D_glrlm_RunEntropy_CDFI_paramsName1

+0.475*gradient_glrlm_ShortRunLowGrayLevelEmphasis_CDFI_paramsName1

-0.473*exponential_firstorder_Minimum_CDFI_paramsName1

-0.470*wavelet-HL_glcm_ClusterShade_US_paramsName1

+0.442*original_firstorder_Minimum_US_paramsName1

-0.406*wavelet-HL_glszm_GrayLevelNonUniformityNormalized_CDFI_paramsName1

+0.380*wavelet-LH_glszm_ZoneVariance_US_paramsName1

+0.378*wavelet-LH_glszm_LargeAreaEmphasis_US_paramsName1

+0.359*lbp-2D_glrlm_RunVariance_CDFI_paramsName1

+0.347*wavelet-HL_firstorder_Energy_CDFI_paramsName1

+0.347*wavelet-HL_firstorder_TotalEnergy_CDFI_paramsName1

+0.333*wavelet-HH_glrlm_RunVariance_US_paramsName1

+0.332*wavelet-HL_ngtdm_Complexity_CDFI_paramsName1

-0.329*wavelet-HH_glcm_Idn_CDFI_paramsName1

+0.317*wavelet-LH_glcm_Imc1_CDFI_paramsName1

-0.301*log-sigma-3-0-mm-3D_glszm_ZonePercentage_CDFI_paramsName1

+0.270*wavelet-HL_glrlm_RunEntropy_CDFI_paramsName1

+0.268*wavelet-HH_glcm_JointEnergy_US_paramsName1

-0.267*logarithm_firstorder_Kurtosis_US_paramsName1

-0.247*gradient_firstorder_Range_CDFI_paramsName1

-0.246*gradient_firstorder_Maximum_CDFI_paramsName1

+0.238*wavelet-LH_firstorder_Skewness_CDFI_paramsName1

-0.212*wavelet-HL_glszm_SmallAreaLowGrayLevelEmphasis_CDFI_paramsName1

+0.211*gradient_glszm_HighGrayLevelZoneEmphasis_US_paramsName1

+0.174*exponential_glszm_ZoneEntropy_US_paramsName1

-0.106*log-sigma-3-0-mm-3D_firstorder_Maximum_CDFI_paramsName1

+0.258
