# Supplementary material for: Dual-modal radiomics ultrasound model to diagnose cervical lymph node metastases of differentiated thyroid carcinoma: a two-center study
Source: Cancer Imaging. 2025 Jan 20;25:4. doi: 10.1186/s40644-025-00825-9 (PMC11749166; doi:10.1186/s40644-025-00825-9)
Supplement: Supplementary file 4 — Supplementary Material 4 [file 40644_2025_825_MOESM4_ESM.docx]

**Supplementary file 4**

**
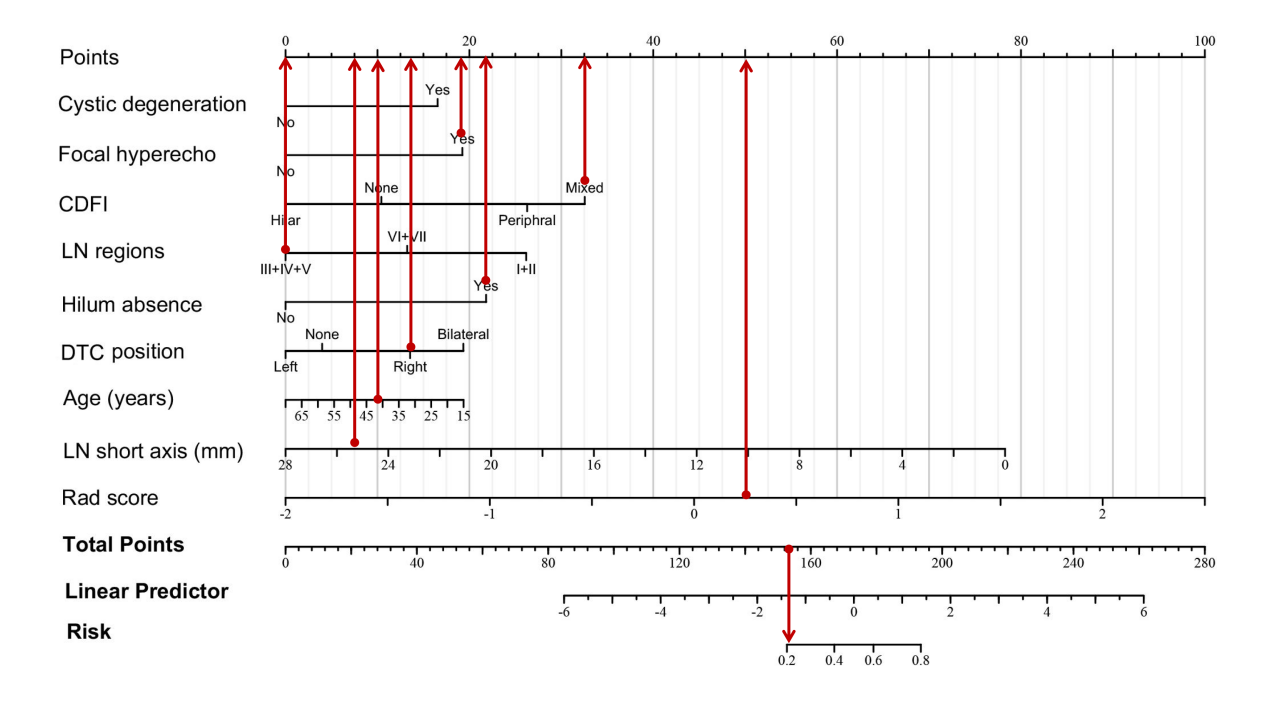
**

**Figure S4. The nomogram for prediction metastatic LNs of a patient with DTC.** A 42‐year‐old patient (10 points) had a primary PTC at right thyroid lobe (13 points). Ultrasound examination showed a suspicious LN at right III region (0 point). The long diameter and short axis of the LN were 95mm and 25mm (8 points), respectively. The LN was absence of hilum (21 points) and presence of focal hyperecho (19 points) inside. The CDFI showed mixed blood flow in LN (33 points). The Rad-score was calculated as 0.5 (50 points). To use the nomogram, the specific points were located on each variable axis. Red lines were drawn upward to determine the points received by each variable; the sum of these points (154 points) was located on the Total Points axis, and a line was drawn downward to Risk axis to determine the probability of metastatic LNs. DTC: differentiated thyroid cancer; LNs: lymph nodes; CDFI: color doppler flow imaging.
